# Geochemistry and tectonic significance of late Paleoproterozoic A-type granites along the southern margin of the North China Craton

**DOI:** 10.1038/s41598-019-56820-1

**Published:** 2020-01-09

**Authors:** Yan Wang, Yi-Zeng Yang, Wolfgang Siebel, He Zhang, Yuan-Shuo Zhang, Fukun Chen

**Affiliations:** 1 Key Laboratory of Crust–Mantle Materials and Environments, School of Earth and Space Sciences, University of Science and Technology of China, Hefei, China; 2 Institute of Earth and Environmental Sciences, Albert-Ludwig University Freiburg, Freiburg, 79104 Germany

**Keywords:** Geochemistry, Precambrian geology, Petrology

## Abstract

The Longwangzhuang pluton is a typical example of Paleoproterozoic A-type granite intrusions at the southern margin of the North China Craton. This pluton is composed of arfvedsonite granite and minor aegirine–augite granites. Samples from both granite types display similar zircon U-Pb ages with ^207^U-^206^Pb ages of 1612 ± 19 Ma [mean square weighted deviation (MSWD) = 0.66] and 1609 ± 24 Ma (MSWD = 0.5), respectively. The granites exhibit similar high silica (SiO_2_ = 71.1–73.4 wt.%), high alkaline (Na_2_O + K_2_O = 8.10–9.26 wt.%, K_2_O/Na_2_O > 1), and low Al_2_O_3_ (11.8–12.8 wt. %) contents and metaluminous to weakly peraluminous bulk chemistry. The chemical variations of the Longwangzhuang pluton suggest the effects of mineral fractionation. In addition, all samples show typical characteristics of A-type granites, such as high 10000Ga/Al ratios (4.10–7.28), high FeO_tot_/(FeO_tot_ + MgO) ratios (0.88–0.99), and high Zr (484–1082 ppm), Ce (201–560 ppm), and Y (78–156 ppm) contents. The ε_Nd_(t) values and the (^206^Pb/^204^Pb)_t_, (^207^Pb/^204^Pb)_t_, and (^208^Pb/^204^Pb)_t_ ratios of the arfvedsonite granite samples vary from −4.6 to –5.3, 15.021 to 17.349, 15.241 to 15.472, and 33.206 to 36.905, respectively, and those for the aegirine–augite granite sample amount at −0.2, 14.421, 15.175, and 33.706. The distinct and variable Nd and Pb isotope values indicate the presence of heterogeneous protoliths. Based on its geochemistry, its low initial Pb isotope ratios, and its enrichment in Nd isotopes, we infer that the Longwangzhuang A-type granite is the partial melting product of basement rocks such as the Taihua Group gneisses accompanied by some involvement of juvenile material from the mantle. Together with published data from other Paleoproterozoic A-type granite plutons exposed at the southern margin of the craton, our findings suggest that these granites had a similar origin. Furthermore, geochemically, they can be divided into two groups: A_2_-type, which formed earlier (~1.8–1.6 Ga), and A_1_-type, which formed later (~1.6–1.5 Ga). Combining this information with the variations in whole-rock Nd and zircon Hf isotopic composition at *ca*. 1.6 Ga, we propose that tectonic transformation from post-orogenic to anorogenic magmatism occurred at the southern margin of the North China Craton at that time.

## Introduction

The North China Craton (NCC) preserves key information on the Archean-to-Proterozoic geological evolution and records numerous important geological events during the Precambrian. The final amalgamation of the eastern and western blocks that occurred along the Trans-North China Orogen at *ca*. 1.85 Ga manifests the formation of the NCC^1^. After the collision, a major transition took place in the evolution of the NCC, and the craton was affected by intensive anorogenic magmatism documented by the formation of aulacogens, mafic dyke swarms, outpourings of volcanic rocks, anorthosite–mangerite–charnockite–granite (AMCG) suites, and A-type granites^2–9^. The Paleo-Mesoproterozoic (2.1–1.2 Ga) period is considered a key period of assembly, growth, and breakup of the Columbia (or Nuna) supercontinent^1,9^. Given the abovementioned magma activities and products, many researchers believe that the NCC was involved in the Columbia supercontinent assembly.

Compared to the northern NCC, Paleoproterozoic magmatism at the southern margin of the NCC is not well understood^10^. The nature of the Paleoproterozoic tectonothermal event along the southern NCC has long been debated. A few studies have been performed on mafic dyke swarms^3,5^, volcanic rocks^9,11^, and alkaline rocks, which are thought to be related through time and space distribution. However, no detailed information is available about this association, and the petrogenesis and tectonic setting remain controversial^12^. A detailed study of the late Paleoproterozoic A-type granites could provide critical insights into the nature of the southern NCC. Some researchers believe that the Xiong’er volcanic rocks developed in a continental rift environment^9^ or in a post-collisional setting^13^, whereas others have argued for a mantle plume origin^11^. Systematic investigation of the Paleo- to Mesoproterozoic magmatism in the NCC could provide insight into the crust–mantle interactions and geodynamic processes that occurred during that time. This information is crucial for a better understanding of the continental growth and evolution of the NCC as well as of the Columbia supercontinent.

Paleo- to Mesoproterozoic alkaline granitoids occur along the southern margin of the NCC. Some of these rocks have A-type affinity, as exemplified by high Ga/Al ratios and enrichment of iron and high field strength elements (HFSEs) and show low Sr contents, suggesting an extensional environment^12,14–17^. Such rocks were rare in the Archean and the early-mid Paleoproterozoic but have been identified extensively worldwide since the late Paleoproterozoic^15,16,18^. The occurrence of Paleoproterozoic A-type granites has significant implications for the geodynamic transition from assembly, growth and breakup of the Columbia supercontinent, which is believed to provide a new perspective on geochemical constraints in the tectonic setting^19–21^. However, little is known about the space–time distribution of the A-type granites in the alkaline rock belt along the southern margin of the NCC and the petrogenetic and geological setting of these A-type granites remains ambiguous.

The Longwangzhuang (LWZ) pluton is the largest Paleo- to Mesoproterozoic A-type granitic intrusion along the southern margin of the NCC. Previous studies focused mainly on the formation time of the pluton^17,22^, but the petrogenesis and the geodynamic setting of the LWZ still have to be investigated in more detail. Some researchers have argued that the LWZ was produced during partial melting of the lower crust in an extensional tectonic environment^17,23^, whereas others have suggested that it was produced by extreme fractional crystallization of an alkali basaltic magma from the enriched mantle^24^. In the present study, we report zircon U-Pb ages, geochemical data, and Sr-Nd-Pb isotopic compositions for the LWZ pluton. Magma source characteristics of the LWZ pluton are discussed based on this data set and on previously published data on zircon Hf and whole-rock O isotope compositions. Taking into account data from other Paleo- to Mesoproterozoic typical A-type granites from the southern NCC, we aim to constrain the petrogenesis of the A-type granites and to understand the tectonic setting of the southern margin of the NCC during the Paleoproterozoic.

## Geological Background

**Figure 1:**
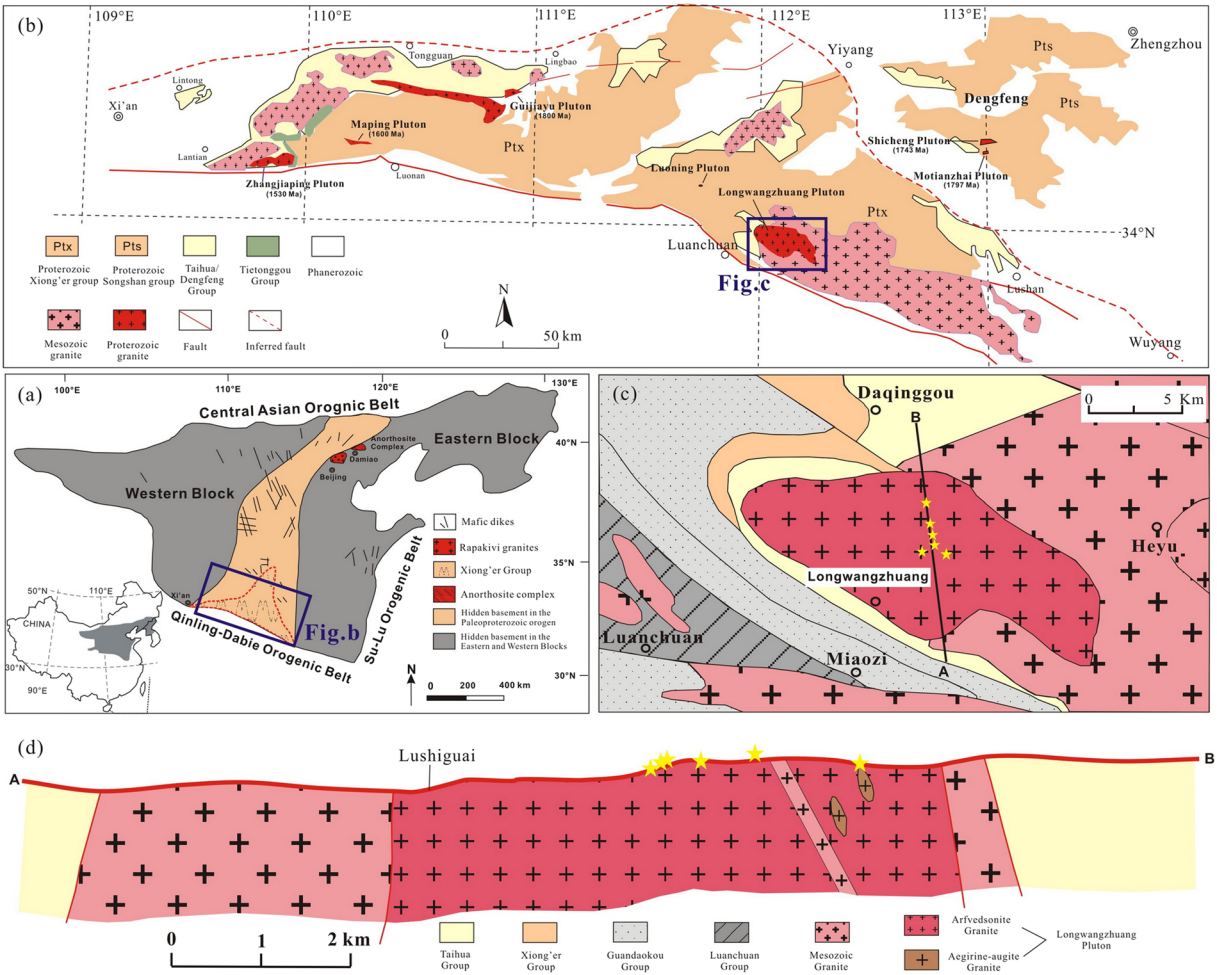
(**a**) Tectonic sketch map of China and simplified geological map of the North China Craton (NCC)^12^; (**b**) Distribution map of the Proterozoic A-type granites in the southern margin of the NCC^52^; (**c**) Geological map of the Longwangzhuang pluton^17^, (**d**) Profile showing the stratigraphic strata and granitic intrusions indicated in (**c**)^17^. The sampling locations are listed in (**c**,**d**).

The growth of the NCC began in the early Archean at about 3.8 billion years ago with the formation of the first continental nuclei. The formation and stabilization of various micro-blocks occurred prior to the late Archean (2.5 Ga). The amalgamation of two major blocks (namely the *western* and *eastern* blocks) along the Trans-North China Orogen occurred at *ca*. 1.85 Ga; this period is thought to be a major cratonization period in the history of the NCC^5,12,25–28^. This amalgamation was followed by rifting, intrusion of mafic dykes, and the formation of A-type granites related to the breakup of the NCC. The rift-related rocks are concentrated in the so-called Xiong’er and Yanshan aulacogens (Fig. 1a). In its present position, the NCC is bordered by the Central Asian Orogenic belt to the north, the Qinling–Dabie–Sulu Orogenic Belt to the south, and the Pacific convergent plate system to the east^28^.

The southern margin of the NCC consists mainly of Neoarchean to early Paleoproterozoic basement rocks and overlying late Paleoproterozoic to Phanerozoic cover sequences^29,30^. The Neoarchean crust to early Paleoproterozoic basement rocks are composed of two distinct tectonic complexes: the Dengfeng Group in the northeast and the Taihua Group in the south (Fig. 1b). The Neoarchean Dengfeng granite–greenstone terrane comprises plutonic rocks and supracrustal assemblages^30^. The Taihua Group consists of amphibolite- to granulite-facies metamorphic rocks that are exposed in the Lushan, Xiaoqinling and Xiong’ershan areas^31^. This rock unit can be divided into the lower and upper Taihua Group. The lower Taihua Group is composed mainly of high-grade sillimanite–garnet gneiss, graphite-bearing gneiss, quartzite, banded iron formations, and marble, with minor mafic granulite, amphibolite, and granitoid rocks^30^, whereas the upper Taihua Group consists predominantly of tonalitic-trondhjemitic-granodioritic (TTG) gneiss with minor supracrustal rocks. The Taihua Group in the Lushan area presents the most complete successions including supracrustal rocks and gneiss series. The Taihua Group in the Xiaoqinling and Lushan areas has been dated at both Archean (~2.9–2.7 Ga) and early Paleoproterozoic (~2.5–2.2 Ga), whereas the Xiong’ershan area with similar gneisses has been dated at ~2.5–2.0 Ga^32,33^.

A substantial amount of late Paleoproterozoic to Mesoproterozoic magmatism also developed along the southern margin of the NCC, including mafic dyke swarms^3,4,11^, volcanic rocks^9,11^, and granite rocks^12,15–17,22–24^. Notably, some of these rocks show an affinity to A-type granite (Fig. 1b), such as the *ca*. 1.8-Ga Motianzhai and Luoning granites^14^, the *ca*. 1.74-Ga Shicheng granite^12^, the *ca*. 1.6-Ga Longwangzhuang and Maping granites^17,22–24,34^, and the *ca*. 1.53-Ga Zhangjiaping granite^15^. The majority of these granites intruded into the TTG gneiss basement or volcanic-sedimentary sequences.

The LWZ pluton is located in the Xiong’ershan–Waifangshan region of the southern margin of the NCC (Fig. 1b). In this region, Archean medium- to high-grade metamorphic rocks from the Taihua Group as well as supracrustal volcanic rocks of the Xiong’er Group (1.80–1.75 Ga) are overlain by undeformed Mesoproterozoic to Phanerozoic supracrustal rocks of the Guandaokou and Luanchuan Groups. Volcanic rocks of the Xiong’er Group consist of basaltic andesites, andesites, rhyolitic lavas, and minor pyroclastic rocks, with an overall thickness of ≥7600 m^35^. The Guandaokou and Luanchuan Groups consist of a marine sequence of clastic and carbonate rocks^36^. The LWZ pluton is located *ca*. 10 km east of Luanchuan Town (Fig. 1b) and covers an outcrop area of *ca*. 140 km^2^ and intrudes gneisses of the upper Taihua Group. Along its eastern side, the pluton is intruded by late Cretaceous Heyu granite (Fig. 1c,d).

## Sample Description

**Figure 2:**
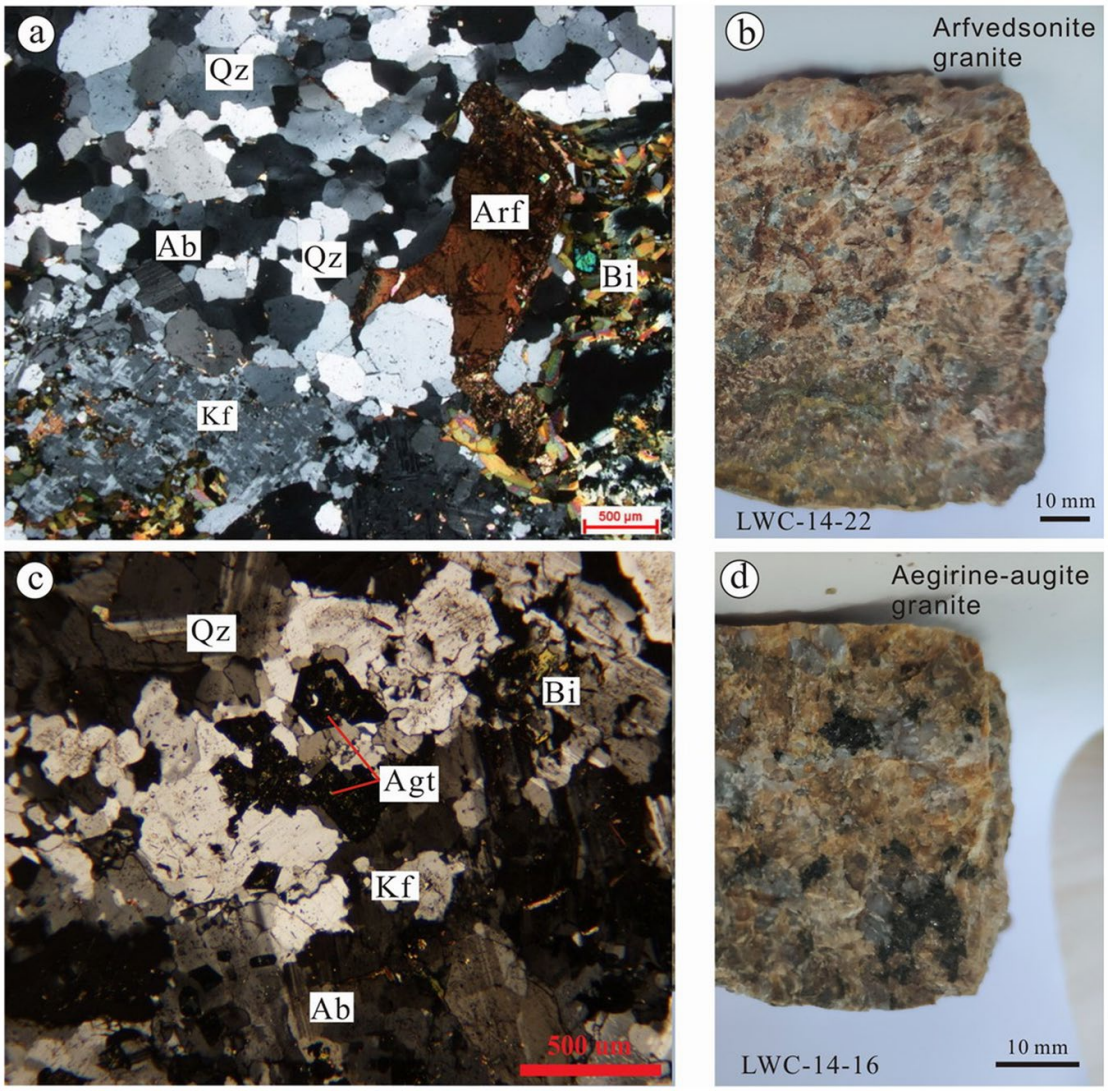
Representative microphotographs of granites under plane-polarized light (**a**,**c**) and hand specimens (**b**,**d**). (**a**) Aegirine–augite granite sample has a mineral assemblage of Kf + Ab + Bt + Qz + Agt; (**c**) arfvedsonite granite samples have a mineral assemblage of Qz + Kf + Bt + Mag + Arf + Ab. Abbreviations: Ab, albite; Agt, aegirine–augite; Arf, arfvedsonite; Bt, biotite; Kf, K-feldspar; Qz, quartz.

Six samples collected from the LWZ pluton were fresh without alteration. As shown in Fig. 1d, arfvedsonite granite is the major rock type in the LWZ pluton, with some aegirine–augite granites in the western margin of the pluton^22,24^. Microphotographs of thin sections showing the textural relationships of the LWZ rocks are presented in Fig. 2. The arfvedsonite granite is dark red in color and coarse-grained in texture (Fig. 2a,b). Modal mineral composition is K-feldspar (40–50 vol.%), quartz (25–35 vol.%), albite (5–10 vol.%), biotite (<5 vol.%) and arfvedsonite (5–10 vol.%). K-feldspar is usually anhedral or semi-euhedral and slightly altered into sericite. Quartz commonly occurs as aggregates with undulate extinction. Arfvedsonite consists of granular or irregular aggregates enwrapping quartz, K-feldspar, and albite. Some grains are replaced by biotite, quartz, and magnetite. Biotites are present as scale-like platy aggregates. The medium-coarse-grained aegirine–augite granite (Fig. 2c,d) is light gray and consists mainly of K-feldspar (55 vol.%), quartz (30 vol.%), albite (5 vol.%), biotite (<5 vol.%), and aegirine–augite (5 vol.%–10 vol.%). K-feldspar is usually anhedral microcline. Quartz commonly occurs as aggregates, and plagioclase is usually anhedral or semi-euhedral. Small amounts of biotite and albite can be observed. Accessory minerals include magnetite, zircon, sphene, apatite, and opaque phases. Late-stage gabbro, syenite, and granite porphyry veins are frequently seen in the pluton.

## Analytical Results

The results and methods used for the zircon U–Pb, geochemical, and Sr–Nd–Pb isotopic analyses are provided as the supplementary information in Tables S1–S4.

### Zircon U-Pb ages

**Figure 3:**
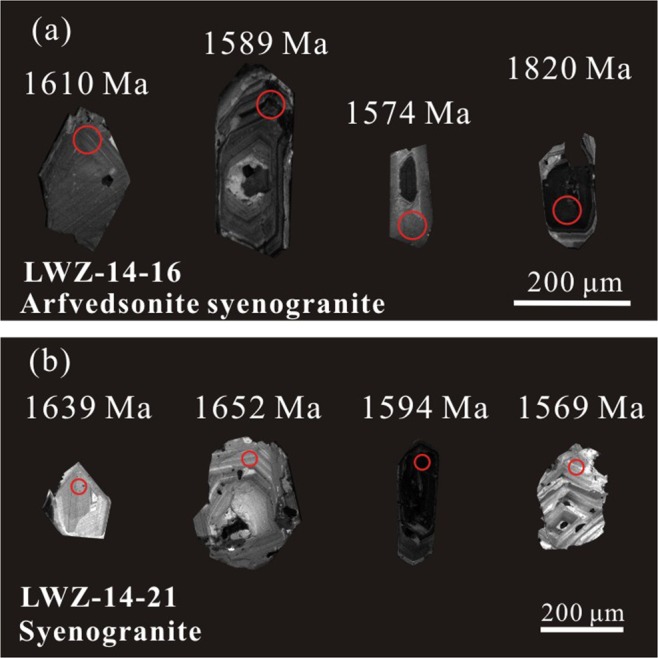
Representative cathodoluminescence images of zircon grains from (**a**) aegirine–augite granite and (**b**) arfvedsonite granite from the LWZ pluton showing ^206^Pb/^207^Pb ages.

**Figure 4:**
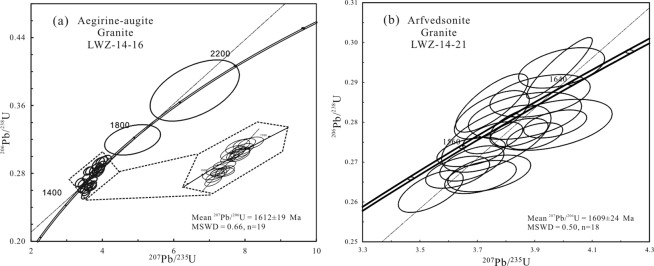
Zircon U-Pb concordia plots of aegirine–augite granite and arfvedsonite granite from the LWZ pluton.

Cathodoluminescence (CL) images of zircon grains from the two granite types are presented in Fig. 3. Most zircon grains from the aegirine–augite granite sample have euhedral crystal shapes and lengths of 200–300 μm. The majority of these zircons are faintly yellow and transparent with typical oscillatory magmatic zoning. Nineteen zircon U-Pb isotopic data spot analyses were obtained from these grains (Fig. 4a). Th and U contents are variable with Th/U ratios ranging from 0.38 to 1.39. Two older zircons with ^207^Pb/^206^Pb ages of 1973 Ma and 1820 Ma were found in this sample. Most dating results show different extents of lead loss, yielding young ^206^Pb/^238^U ages. These analyses define a coherent age group with an average ^207^Pb/^206^Pb age of 1612 ± 19 Ma (MSWD = 0.66).

Zircons of the arfvedsonite granite sample resemble those of the aegirine–augite granite sample (Fig. 3). However, no older zircons were found in the arfvedsonite granite. Eighteen spot analyses were obtained with Th/U ratios ranging from 0.35 to 1.11. The majority of these grains show lead loss to different extents and give consistent ^207^Pb/^206^Pb ages of 1609 ± 24 Ma (MSWD = 0.5) (Fig. 4b). These ages are consistent with the published zircon ^207^Pb/^206^Pb ages of 1625 ± 16 Ma obtained by sensitive high-resolution ion microprobe (SHRIMP)^22^ and 1602 ± 7 Ma^24^ and 1616 ± 20 Ma^17^ obtained by LA-ICP-MS. This finding suggests that the two types of granite sampled from the LWZ pluton were formed contemporaneously, and ~1.6 Ga represents the crystallization age of the pluton.

### Whole-rock geochemistry

**Figure 5:**
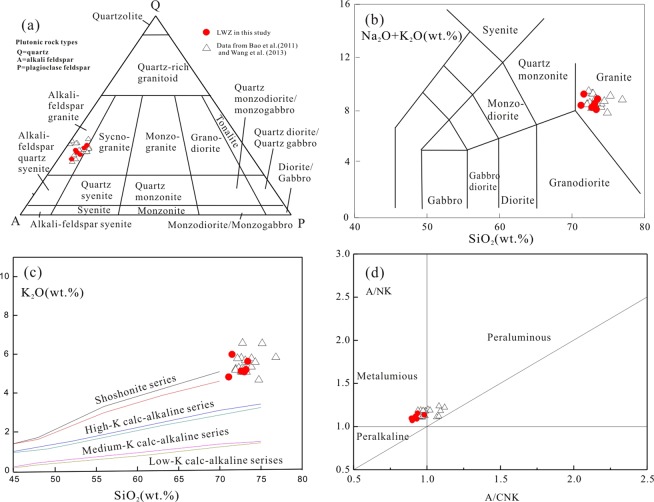
Major-element geochemical plots for the LWZ pluton: (**a**) QAP (Q, quartz; A, alkali feldspar; P, plagioclase) normative diagram^72^; (**b**) Total alkali versus silica (TAS) diagram^73^; (**c**) SiO_2_ and K_2_O correlation diagram^74^; (**d**) A/NK [A, aluminium oxide; N, sodium oxide; K, potassium oxide; molar Al/(Na + K)] versus A/CNK [A, aluminium oxide; C, calcium oxide; N, sodium oxide; K, potassium oxide; molar Al/(Ca + Na + K)] diagram^75^. Data are colletcted from references^17,27^ and the present study.

**Figure 6:**
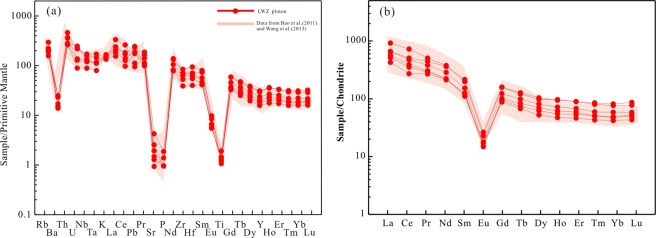
(**a**) Primitive mantle–normalized trace element and (**b**) chondrite-normalized rare earth element (REE) diagrams. Normalized values are from the literature^76^.

In the Streckeisen diagram the LWZ samples plot into the alkali feldspar quartz syenite and alkali feldspar granite fields (Fig. 5a). The granite have high silica (SiO_2 = _71.1 to 73.4 wt. %) and alkali contents (Na_2_O + K_2_O = 8.10 to 9.26 wt.%, K_2_O/Na_2_O > 1) and low Al_2_O_3_ (11.8 to 12.8 wt. %) and MgO (0.06 to 0.22 wt.%) contents and range from alkali-calcic to alkalic with high FeO_tot_ (1.56 to 4.02 wt.%) and high (Na_2_O + K_2_O)/Al_2_O_3_ ratios (0.68 to 0.72). In the SiO_2_ versus Na_2_O + K_2_O (TAS) diagram, all samples fall in the field of granite (Fig. 5b). In the SiO_2_ versus K_2_O diagram (Fig. 5c), the samples show a high-K calc-alkaline to shoshonite trend. The LWZ samples are metaluminous to weakly peraluminous with aluminum saturation index [ASI; molar Al_2_O_3_/(Na_2_O + K_2_O + CaO)] values between 0.93 and 0.98 (Fig. 5d). In the primitive mantle–normalized trace elements diagram (Fig. 6a), samples from the LWZ pluton are significantly enriched in large ion lithospheric elements (LILE) and REE (ƩREE + Y of 1152 to 2195 ppm) but depleted in P and Ti; the samples also show slightly negative Nb-Ta anomalies. Chondrite-normalized REE patterns for the rocks are similar to each other with strong negative Eu anomalies (Eu/Eu* < 0.2) and fractionation between light and heavy REEs (Fig. 6b).

### Whole-rock Sr-Nd-Pb isotopic compositions

The granites from the LWZ pluton have low and variable initial ^87^Sr/^86^Sr ratios ranging from 0.5382 to 0.7151. The large scatter of the calculated variable initial ^87^Sr/^86^Sr ratios is possibly due to the large error caused by their high Rb/Sr ratios (5.8–27.5). All rocks show limited variation in their radiogenic Nd and Pb isotope compositions when calculated back to 1600 Ma. The arfvedsonite granite samples have almost the same initial Nd isotopic compositions, which mainly vary from 0.510301 to 0.510332; however, the aegirine–augite granite sample has a higher ^143^Nd/^144^Nd ratio of 0.510561. The arfvedsonite granite samples show similar ε_Nd_(t) values ranging between –4.6 and –5.2; a higher ε_Nd_(t) value of –0.2 was obtained for the aegirine–augite granite sample. The calculated Nd two-stage model ages (T_DM2_) fall within the range of 2.47 to 2.80 Ga.

The analyzed samples have relatively variable present-day Pb isotopic compositions with ^206^Pb/^204^Pb ratios of 18.00–20.03, ^207^Pb/^204^Pb ratios of 15.54–15.73, and ^208^Pb/^204^Pb ratios of 39.52–48.28. The calculated initial Pb isotope compositions of the arfvedsonite granite samples vary from 15.03 to 17.35 for ^206^Pb/^204^Pb(t), from 15.24 to 15.47 for ^207^Pb/^204^Pb(t), and from 33.21 to 36.39 for ^208^Pb/^204^Pb(t). The corresponding values of the aegirine–augite granite sample are 14.42, 15.18, and 33.71, respectively. The low radiogenic Pb isotopic composition of the LWZ pluton matches the Pb isotopic features of the basement rocks of the NCC in the Paleoproterozoic.

## Discussion

### Formation age and petrogenesis of the LWZ pluton

Zircon U-Pb dating of the arfvedsonite granite and aegirine–augite granite samples of the LWZ yields similar Paleoproterozoic ages and confirms the findings of previous studies that the LWZ pluton intruded at *ca*. 1600 Ma. Older zircons in the aegirine–augite granite sample with ^207^Pb/^206^Pb ages ranging from 1.7 to 2.0 Ga (Figs. 3a and 4a) may have been inherited from the ca. 1.78 billion year old Xiong’er Group which consists of rift-related volcanic rocks.

**Figure 7:**
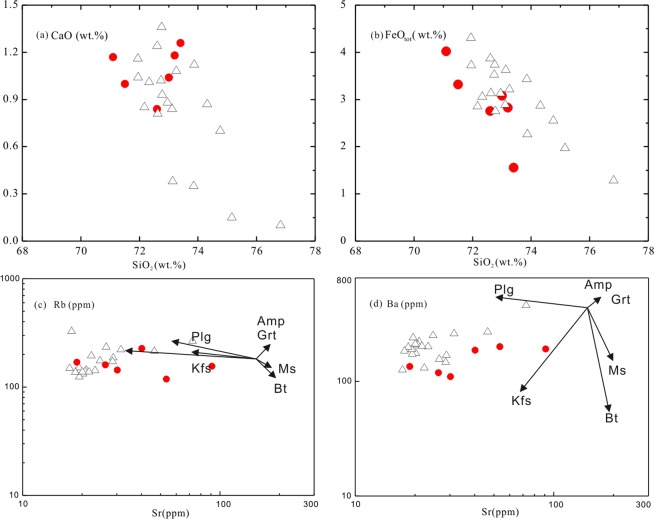
Chemical discrimination diagrams illustrating the mineral fractionation process for the LWZ pluton. (**a**) SiO_2_ versus CaO; (**b**) SiO_2_ versus FeO_tot_; (**c**) Rb versus Sr^77^; (**d**) Ba versus Sr^77^. Abbreviations: Bt, biotite; Cpx, clinopyroxene; Hbl, hornblende; Kf, K-fekdspar; Plg, plagioclase. Symbols and data sources as in Fig. 5.

In the trace element spider diagram the LWZ samples show pronounced enrichment in LILEs, such as Rb and Th, and moderate enrichment in high field strength elements (HFSE), and depletion in Ba, Sr, Ti, and P (Fig. 6a). As shown in Fig. 6b, the chondrite-normalized REE patterns are smoothly right-inclined with strong negative Eu anomalies (Eu/Eu* = 0.12 to 0.16). The slightly negative Nb, Ta, and Ti anomalies are believed to have resulted from fractionation of Ti-bearing phase, and the negative P anomalies from apatite separation. Low Eu/Eu* values require extensive fractionation of plagioclase, K-feldspar, or both. The major element concentrations of the LWZ pluton are roughly negatively correlated with SiO_2_ contents (e.g., Fig. 7a,b). The negative correlation of CaO (0.10–1.36) and FeO_tot_ (1.56–4.02) contents with varying SiO_2_ contents indicates fractionation of feldspar and Fe-Ti oxides. In Fig. 7, negative correlations of Rb and Ba with varying Sr concentrations (19–91 ppm) suggest crystallization of K-feldspar and plagioclase.

**Figure 8:**
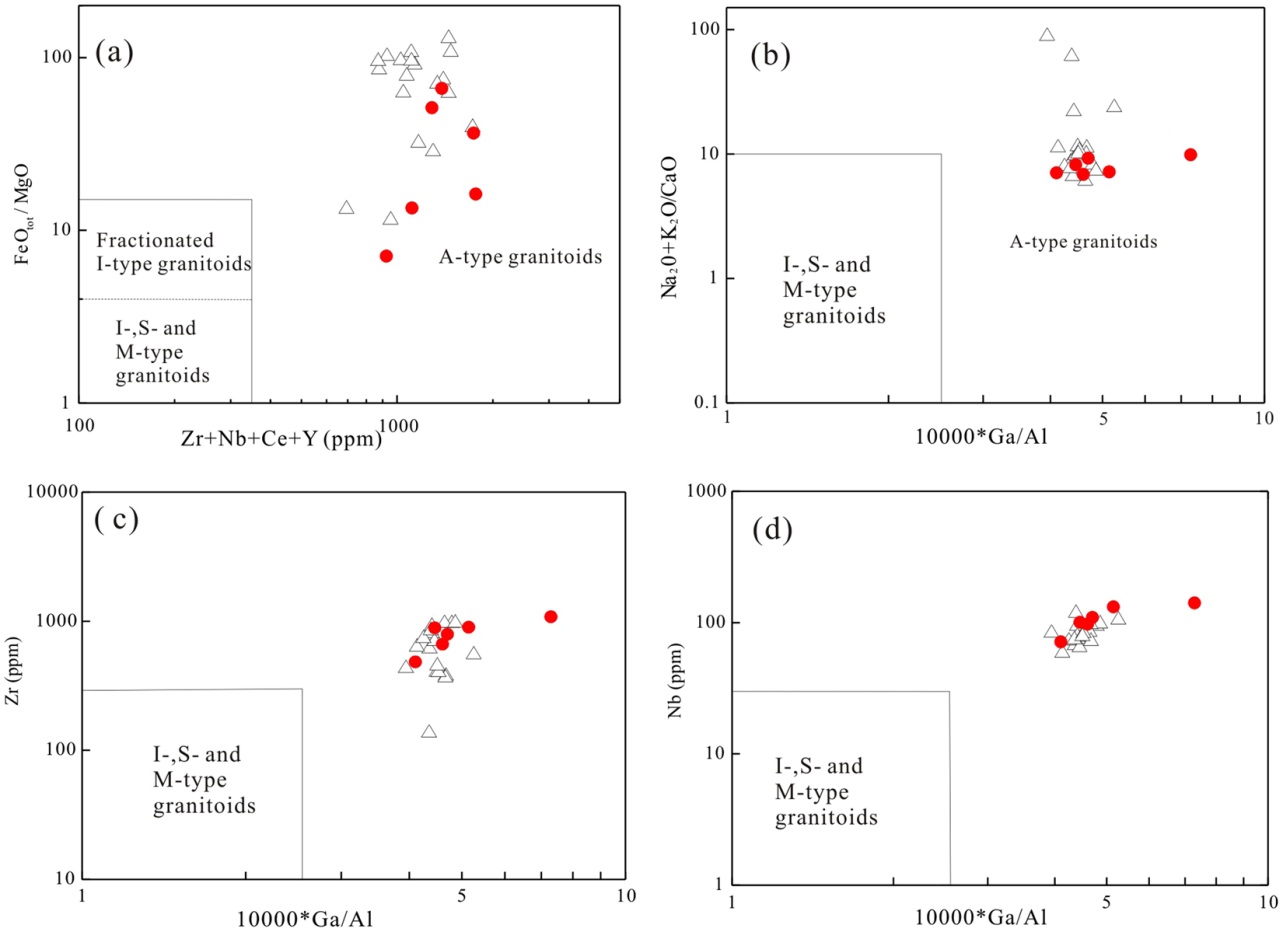
Geochemical classification for the LWZ A-type granites^18^. (**a**) Zr + Nb + Ce + Y versus FeOt/MgO diagram (FG, fractionated felsic granites; OGT, unfractionated M-, I-, and S-type granites); (**b**) 10000Ga/Al versus K_2_O + Na_2_O diagram; (**c**) 10000Ga/Al versus Zr diagram; (**d**) 10000Ga/Al versus Nb diagram.

**Figure 9:**
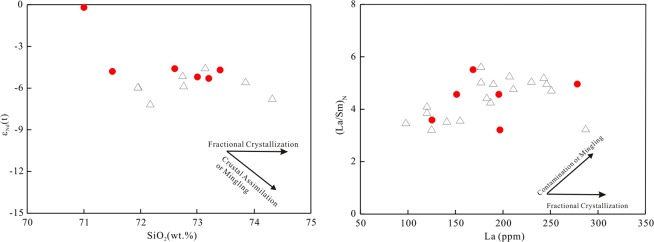
(**a**) SiO_2_ concentration versus ε_Nd_(t) value and (**b**) La contents versus (La/Sm)_N_ ratios for the LWZ pluton. Symbols as in Fig. 5.

The LWZ pluton is composed of alkaline minerals (e.g., arfvedsonite and aegirine–augite) and enriched in HFSE, and thus resembling typical A-type granites (Fig. 2). However, perthites, which form by high-temperature and low-pressure crystallization^15,37^, share similar features to A-type granite. The 10000Ga/Al ratios for the LWZ range from 4.10 to 7.28; these ratios are higher than the global average value (3.75) of A-type granites^18^. Based on their high Zr (484–898 ppm), Ce (201–560 ppm), and Y (78–148 ppm) contents and high 10000Ga/Al ratios, the samples fall in the field of A-type granites in geochemical classification diagrams (Fig. 8a–d). As shown in Fig. 9a, the ε_Nd_(t) values of the LWZ granites are distinct and show slightly negative correlations with increasing SiO_2_. Combining this information with the slight increase between the ratios of (La/Sm)_N_ and La content (Fig. 9b), we suggest that the LWZ pluton experienced some contamination or a magma mingling during formation.

A-type granites have been recognized as a distinct group of granites for nearly 40 years^19^. One challenge is distinguishing A-type granites produced predominantly by extreme fractional crystallization of a mantle derived mafic magma, or those generated by partial melting of crustal sources, or by mixing of the two end members^7,38^. Until now, melting experiments have only partially achieved magma with similar major and trace element features to those of A-type granites (e.g., low Al, Ca, Mg, Sr, Eu contents; high Ga/Al ratios) via a combined process of partial melting of lower crustal lithologies (i.e., tonalite, granodiorite, charnockite, and granulitic residuum) followed by fractional crystallization^39–42^. However, no convincing A-type liquids were produced experimentally solely by crustal materials; thus, it is reasonable to infer some involvement from mantle-derived melts^19^. Moreover, some researchers have noted that the importance of pressure and temperature conditions in the genesis of A-type granite should be emphasized in addition to the composition of the source rock^40^.

Paleoproterozoic mafic rocks have been recognized at the southern margin of the NCC (zircon U-Pb age: 1819 ± 10 Ma^43^) and show similar ε_Nd_(t) values (–5.5 to –0.6^44^) to those of the LWZ pluton; however, the low initial Pb isotope ratios reported in the present study [14.42–17.35 for ^206^Pb/^204^Pb(t), 15.18–15.47 for ^207^Pb/^204^Pb(t), and 33.21–36.39 for ^208^Pb/^204^Pb(t)] suggest a crustal source of the LWZ magma. Previously reported high whole-rock oxygen isotope values (δ^18^O = 11.3‰)^23^ also support a crustal origin. Moreover, considering the low Mg contents (0.05–0.22 wt.%) and low Cr (3.87–5.33 ppm) and Ni (0.27–1.82 ppm) concentrations, the LWZ pluton presents crustal characteristics, which cannot be easily explained by fractional crystallization of early-formed mafic rocks.

The proposed crustal source rocks of A-type granites that have been favored are from the lower crust, including (1) metasedimentary rocks, (2) granulitic residuum from a melt of previously extracted I-type granite magmas, and (3) calc-alkaline granitoids, such as tonalite and granodiorite^18,40,45–47^. The metasedimentary rocks typically have low alkaline but high aluminous contents and exhibit peraluminous character^48,49^. The high alkaline contents and lower aluminous contents with metaluminous to weakly peraluminous character of the LWZ pluton argue against a metasedimentary origin. Additionally, experimental results show that partial melting of the refractory residue of a granulite meta-igneous source that had been previously depleted in a hydrous felsic melt would produce granitic magma depleted in alkalis relative to alumina and in TiO_2_ relative to MgO^39,40^. However, the LWZ pluton is characterized by high TiO_2_/MgO ranging from 1 to 12^17^, and thus cannot be explained by a residual granulite origin. More importantly, experimental petrology has established that a residual granulitic source is unlikely to generate A-type granitic melts because it is too refractory^39^. The high Rb/Sr ratios (5.8–27.5) of the LWZ pluton also differ from the residual source composition left behind from the generation of I-type granite^39^. In contrast, the geochemical features of the LWZ pluton [e.g., high K_2_O/Na_2_O ratios (1.35–1.83), low P_2_O_5_ (0.02–0.04 wt.%) contents, depletion in Eu and Sr] are more similar to those of the experimental melt derived through partial melting of lower crust, such as tonalite and granodiorite, at high temperature.

With respect to the occurrence of mafic minerals, such as arfvedsonite, aegirine and augite, the LWZ pluton is similar to traditional A-type granites^47^. The metaluminous to weakly peraluminous character of the LWZ may be related to partial melting of tonalite and granodiorite with a plagioclase-rich residue at high temperature (950°C) at a shallow crustal level^15,16^. Widespread exposure of the basement rocks of the Taihua Group has been suggested as a potential source for the LWZ pluton^17,23^. The zircon ε_Hf_(t) values of the Taihua Group vary from −5.8 to −7.0^32,50^. The low zircon ε_Hf_(t) values (−17.4 to −8.8^51^) of the Xiong’er Group rule them out as the possible magma source of the LWZ pluton. In the Xiong’ershan area, the Taihua Group is composed of gray gneisses with minor amphibolites^52–54^. The former have positive zircon ε_Hf_(t) values ranging from 0.6 to 9.0, and the latter have zircon ε_Hf_(t) values ranging from −5.0 to 1.2^55^. As shown in Fig. 9, the zircon ε_Hf_(t) values and the whole-rock ε_Nd_(t) values of A-type granites at the southern NCC mostly plot in the evolution field of the Taihua Group, indicating that they were most likely derived from partial melting of basement rocks like those of the Taihua Group^12,14–16,34^. The zircon ε_Hf_(t) values of the LWZ pluton vary between –6.4 and –1.1, and the Hf model ages fall within the range of 2.4–2.6 Ga^17,24^. The whole-rock ε_Nd_(t) values of the LWZ pluton vary from –5.3 to –0.2, and the Nd model ages of the LWZ mostly range from 2.5 to 2.8 Ga, similar to the formation age of the lower NCC crust^55^. Figure 9 shows that both the zircon Hf and whole-rock Nd isotopes of the NCC are slightly more depleted than the Taihua Group rocks, indicating that some juvenile materials had been involved in the generation of this A-type granite.

In summary, the geochemical characteristics of the LWZ granites imply that the magma was generated by partial melting of old crustal source regions, such as the Taihua Group, with some involvement of juvenile mantle materials. The LWZ pluton displays distinct A-type geochemical features, and its time of emplacement can be constrained to the time period of extension in the southern margin of the NCC.

### A-type granites along the southern margin of the NCC

As shown in Fig. 1a, Paleo- to Mesoproterozoic A-type granites from the southern margin of the NCC include the Motianzhai (*ca*. 1.8 Ga), Guijiayu (*ca*. 1.8 Ga), Luoning (*ca*. 1.78 Ga), Shicheng (*ca*. 1.74 Ga), Maping (*ca*. 1.6 Ga), and Zhangjiaping (*ca*. 1.53 Ga) plutons (see supplementary information Table S5). All these plutons were emplaced between *ca*. 1.8 Ga and *ca*. 1.5 Ga^12,14–16,34^. Moreover, several typical A-type granites at the northern NCC are also considered here, such as the Miyun A-type granite and the Shachang rapakivi granite^2,6–8^.

**Figure 10:**
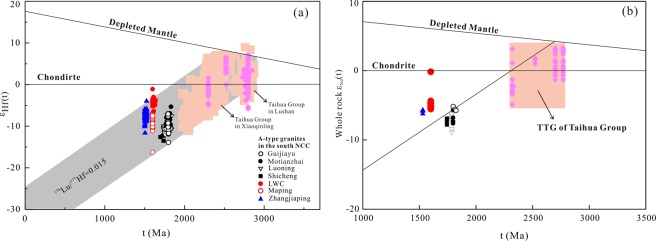
(**a**) Zircon ages versus ε_Hf_(t) and (**b**) ε_Nd_(t) values of Proterozoic A-type granites in the southern margin of the NCC.

Late Paleo- to early Mesoproterozoic A-type granites at the southern margin of the NCC have variable isotope compositions and geochemical characteristics. As shown in Fig. 10a, zircons from these granites have strongly negative ε_Hf_(t) values that are significantly lower than those of mantle-derived rocks^9^ or of newly formed crust-derived rocks^12^, which normally show positive zircon ε_Hf_(t) values. Nd isotopes of these granites plot along the evolution trend of the TTG rocks of the Taihua Group. Therefore, most of the Paleo- to Mesoproterozoic A-type granites at the southern margin of the NCC were likely generated by partial melting of Neoarchean crustal material similar to that of the Taihua Group.

**Figure 11:**
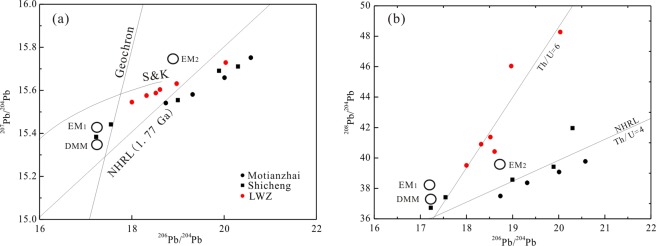
207Pb/^204^Pb versus ^206^Pb/^204^Pb and ^208^Pb/^204^Pb versus ^206^Pb/^204^Pb for LWZ A-type granites. Crustal lead evolution (S&K) and Northern Hemisphere Reference Line (NHRL) are from the literature^78,79^. Data for the Shicheng and Motianzhai A-type granites are from the literature^12^ and are listed in Table SA.

Granites from different sources would show distinct Pb isotopic compositions^56^. The Pb isotopic ratios of the Motianzhai, Shicheng, and LWZ plutons (Fig. 11) conform to the features of the basement rocks in the NCC, suggesting that their source rocks were mostly extracted from the U- and Th-enriched, epi-metamorphic basement^57^. The low initial radiogenic Pb isotopic composition of these A-type granites indicates that the magmas for these granites were mainly derived from crustal basement materials (Fig. 11a). Moreover, the correlation between the ^208^Pb/^204^Pb and ^206^Pb/^204^Pb ratios indicates the heterogeneity of the magma sources of these A-type granites at the southern margin of the NCC (Fig. 11b).

The Paleo- to Mesoproterozoic Maping, Zhangjiaping, Guijiayu, Shicheng, Motianzhai, and LWZ plutons form a continuous A-type granite belt along the southern margin of the NCC. Together they document the youngest extensional-related event in this region. The generation of A-type rocks requires high temperatures^58^, which can be realized by underplating of mantle-derived mafic magmas or upwelling of asthenospheric mantle material. The existence of a mantle plume at that time could explain this magmatism as well as the contemporaneous lithospheric instabilities and rifting processes.

### Geological setting and implications

A-type granites are enigmatic not only with regard to their petrogenesis but also in terms of their tectonic setting and their overall significance in the evolution of the Earth’s lithosphere^59^. Although A-type granites were originally thought to form in rift zones or in stable continental blocks, it is generally accepted that they can both form in post-orogenic and anorogenic settings, such as during lithospheric extension, continental rift formation, and during late- to post-orogenic gravitational collapse following an episode of crustal thickening^18–20,60^. Therefore, understanding the generation of the A-type granites in the southern NCC would provide critical insight into the tectonic evolution and the deep geodynamic processes that occurred during the Paleo-Mesoproterozoic Era.

A-type granites can be subdivided into A_1_ and A_2_ groups based on geochemical composition^21^. The A_1_ group represents magmas emplaced in continental rifts or during intraplate magmatism, whereas the A_2_ group represents magmas derived from continental crust or underplated crust that originated through a cycle of continent–continent collision magmatism^21^. However, it is not easy to distinguish between the two A-type granites owing to the similarity of their features regarding lithology, mineralogy, and geochemistry^61,62^.

**Figure 12:**
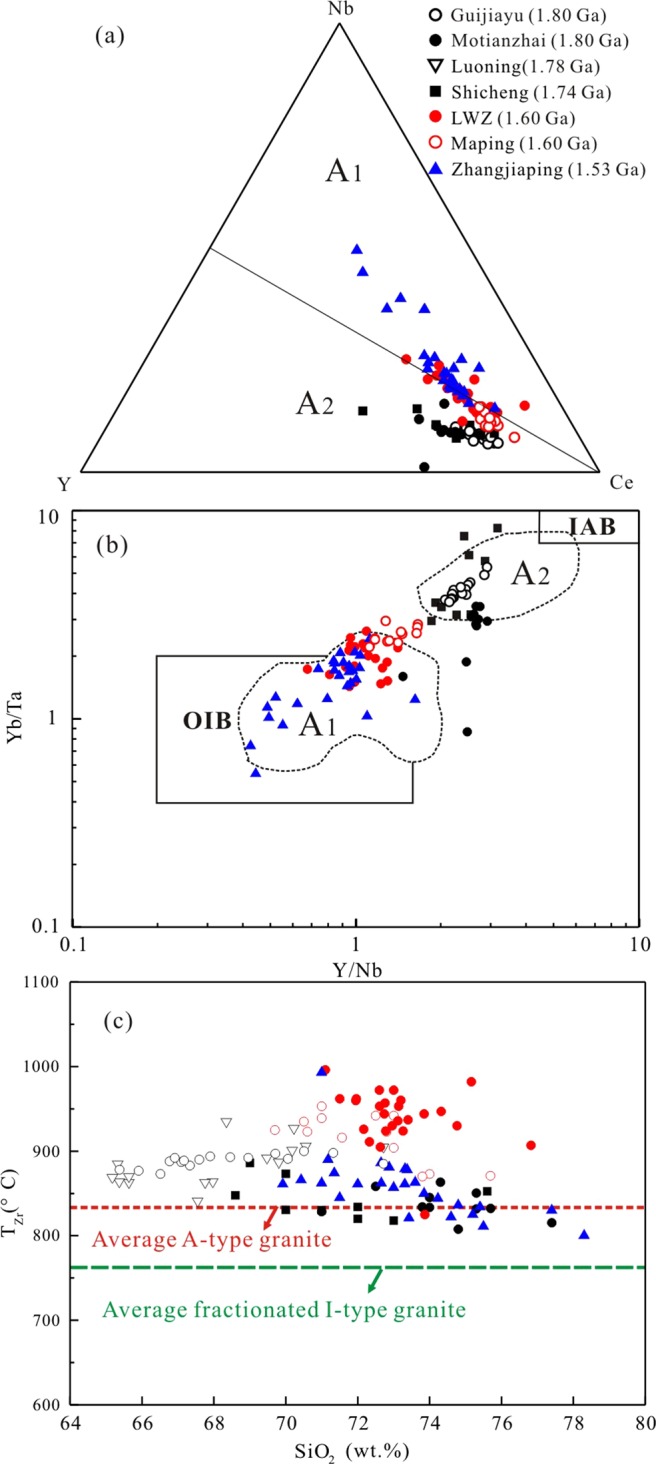
Representative plots for distinguishing between A_1_ and A_2_ granitoids. (**a**) Nb-Y-Ce diagram^21^, (**b**) Yb/Ta versus Y/Nb diagram^60^, (**c**) T_Zr_ (°C) versus SiO_2_ (wt.%) concentration, showing distinction among the Paleo- to Mesoproterozoic A-type granites in the southern margin of the NCC. Abbreviation: T_Zr_ (°C), calculated Zr saturation temperatures. Lines for average A-type granite and fractionated I-type granite are from the literature^47^. Detailed literature data are listed in Table SA.

**Figure 13:**
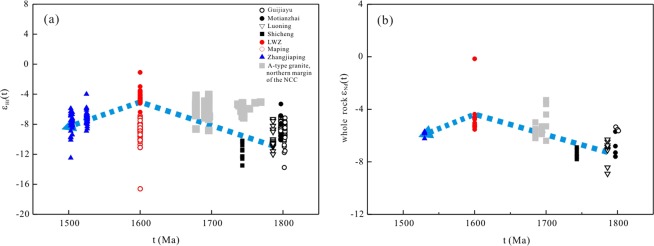
(**a**) ε_Hf_(t) values and (**b**) ε_Nd_(t) values versus zircon ages for the LWZ pluton. Data for the Taihua Group are from the literature^32,33,50,55,80^ and are listed in Table SA.

As shown in Fig. 12a, A-type granites at the southern NCC define a trend from the group A_2_ to the group A_1_ field with decreasing age. A-type granites with formation age >1.74 Ga (Guijiayu, Motianzhai, and Shicheng plutons) fall into group A_2_, whereas the 1.53-Ga Zhangjiaping pluton falls into group A_1_. A similar trend also exists in the Yb/Ta versus Y/Nb diagram (Fig. 12b). In Fig. 12b, the granites show a trend from the island arc basalt (IAB) field to the ocean island basalt (OIB) field with decreasing age. This suggests that that granites forming in different periods were controlled by different tectonic settings. The *ca*. 1.6 billion years old LWZ and Maping A-type granites belong to group A_1_ and group A_2_ (Fig. 12a,b) and show an affinity to within-plate granites in the tectonic discrimination plots (Fig. 13).

Based on Zr saturation thermometry^63^, the crystallization temperatures of the LWZ pluton was high, ranging from 972 °C to 996 °C, higher than the average value of 839 °C reported for A-type granites^47^ (Fig. 12c). Moreover, all of the Paleoproterozoic A-type granites, particularly those of the LWZ and Maping plutons, present high Zr saturation temperatures.

The older A-type granites of the Motianzhai, Guijiayu, Shicheng, and Luoning plutons (>1.74 Ga) have low ε_Hf_(t) values of –13.9 to –5.3^12,14,15^. A-type granites younger than *ca*. 1.6 Ga (e.g., Zhangjiaping) also display relatively low ε_Hf_(t) values for zircon^17,24^ (Fig. 13a). However, A-type granites of *ca*. 1.6 Ga exhibit noticeably larger variations of zircon ε_Hf_(t) values, ranging from –6.4 to –1.1 for the LWZ pluton and from –16.7 to –6.9 for the Maping pluton^34^ (Fig. 13a). A similar phenomenon can also be observed from Nd whole rock systematics (Fig. 13b): the LWZ pluton displays a stronger depletion in Nd isotopic composition than the other A-type granites. Thus, we propose that the 1.6 billion years old A-type granites record a tectonic transformation event during the Paleo-Mesoproterozoic Era.

The geological evolution of the NCC during the early Precambrian remains controversial. One opinion holds that the amalgamation of the NCC occurred at *ca*. 2.5 Ga^64^, whereas others support a model that the western and eastern blocks finally amalgamated by continent–continent collision at *ca*. 1.85 Ga^26,27,65,66^. However, there is general agreement that the NCC was subjected to extensional tectonics during the late Paleoproterozoic and early Mesoproterozoic. Anorogenic events, such as rifting, intra-plate magmatism, and mafic dyke swarms, are widely manifested during this period. Plutonic and volcanic activity related to extensional tectonics in the NCC culminated at 1.8–1.6 Ga. However, knowledge about the tectonic processes associated with the crustal extension of the NCC is very limited. Extension and Paleo-Mesoproterozoic magmatism in the NCC is related either to post-collisional processes such as slab delamination or to the involvement of a mantle plume. The western and eastern blocks are believed to have combined at *ca*. 1.85 Ga, after which the tectonic environment changed from compression to extension^9,67^. Another school of thought argues that the NCC was characterized by orogenic processes at *ca*. 1.9 Ga and post-orogenic rifting events at *ca*. 1.7 Ga, and that the rifting was initiated by mantle plume^17,68,69^.

**Figure 14:**
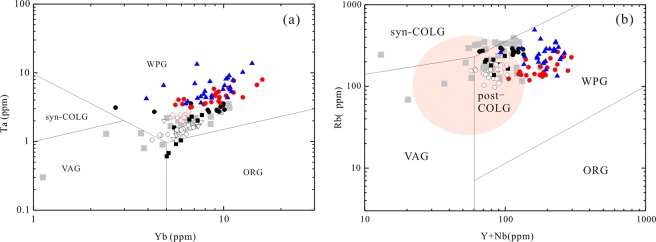
(**a**) Rb concentration versus (Y + Nb) concentration^81^ and (**b**) Ta versus Yb^82^ for a summary of the early Proterozoic A-type granties in the NCC. Abbreviations: ORG, ocean-ridge granite; post-COLG, post-collisional granite; syn-COLG, syn-collisional granite; VAG, volcanic arc granite; WPG, within-plate granite. Symbols as in Fig. 12. Summary data for early Proterozoic A-type granties of the NCC are from the literature^2,6–8,12,14–17,24,34^ and are listed in Table SA.

In tectonic discrimination diagrams (Fig. 14), all A-type granites at the southern margin of the NCC (1.8 to 1.5 Ga) fall in the field of within-plate granites, likely indicating the existence of a continuous extension environment. Post-collisional (1.80–1.68 Ga) and anorogenic (1.60–1.53 Ga) magmatic events suggest that the NCC was in a long-term extensional tectonic setting. The Guijiayu and Motianzhai plutons (~1.8 Ga in age) are characterized by high-K calc-alkaline granitoids, which is typical for granites from continental collision orogenic belts, particularly at the end of the collision^70^, implying a post-collisional extension environment rather than an anorogenic regime^12,15,16^. A-type granites from the northern NCC, such as the 1.75-Ga Changsaoying, 1.70-Ga Shachang, 1.7-Ga Wenquan, and 1.68-Ga Miyun plutons, formed in a post-collisional setting and suggest a tectonic model of continental collision between the western and eastern blocks of the NCC at c*a*. 1.85 Ga^8^. The 1.74-Ga Shicheng granite with group A_2_ features is comparable to the A-type granites of the northern NCC, suggesting a post-orogenic setting^12^. These occurrences indicate that granitic magmatism was widespread throughout the NCC and that strong post-orogenic extension was likely the main reason for the generation and emplacement of the *ca*. 1.8- to 1.68-Ga A-type granites. The LWZ pluton is considered the largest anorogenic intrusion at the southern margin of the NCC^17,22–24^. The LWZ and Maping plutons have high and variable ε_Hf_(t) values and the highest zircon saturation temperatures amongst the A-type granites, likely indicating a tectonic transformation at *ca*. 1.6 Ga.

During the Paleo- to Mesoproterozoic Era, the Columbia supercontinent is thought to have been the Earth’s largest landmass. The fragmentation of Columbia was reported to have begun at *ca*. 1.6 Ga in North China, India, and North America, and the rifting continued until approximately 1.4 Ga in most parts of the supercontinent^70,71^. The final breakup of the Columbia supercontinent was marked by the emplacement of 1.35- to 1.21-Ga mafic dyke swarms in major cratonic blocks throughout the world^9^. The geological setting of the southern NCC during the transition period of the Paleoproterozoic to the Mesoproterozoic is consistent with the evolution of the Columbia supercontinent. The formation of A-type granites may be related to the breakup of the Columbia supercontinent at the end of the Paleoproterozoic. Taking into account the high Zr saturation temperatures observed for the LWZ pluton (972–996 °C, this study) and for the Maping pluton^34^ (870–953 °C), it is reasonable to propose that a mantle plume provided the heat source for partial melting of Archean basement rocks to produce these A-type granites at *ca*. 1.6 Ga. On a broader scale, we tentatively suggest that the A-type granitoids exposed in the NCC were closely related to lithospheric thinning caused by mantle plumes, leading to partial melting of underlying old crustal rocks during the breakup of Columbia. The existence of these A-type granites strongly suggests that the NCC was part of the Columbia supercontinent.

## Conclusions

The LWZ pluton was emplaced at *ca*. 1.6 Ga and exhibits the chemical composition of A-type granites. Radiogenic isotope ratios suggest that the magma was originated by partial melting of old crustal rocks from the NCC with the involvement of some juvenile materials. Combined with evidence from the late Paleo- to early Mesoproterozoic A-type granites distributed along the southern margin of the NCC, these findings suggest a prolonged history of extensional magmatism from 1.80 Ga to 1.53 Ga in this region. Nevertheless, geochemical distinction between these A-type rocks points to a tectonic transformation event at *ca*. 1.6 Ga that is likely related to the breakup of the Columbia supercontinent.

## Supplementary information

Table S1.

Table S2.

Table S3.

Table S4.

Table S5.

Table SA.
