# Supplementary material for: Geochemistry and tectonic significance of late Paleoproterozoic A-type granites along the southern margin of the North China Craton
Source: Sci Rep. 2020 Jan 9;10:86. doi: 10.1038/s41598-019-56820-1 (PMC6952446; doi:10.1038/s41598-019-56820-1)
Supplement: Supplementary file 1 — Table S1. [file 41598_2019_56820_MOESM1_ESM.docx]

**Geochemistry and tectonic significance of late Paleoproterozoic A-type granites along the southern margin of the North China Craton**

**Yan Wang, Yi-Zeng Yang, Wolfgang Siebel, He Zhang, Yuan-Shuo Zhang, Fukun Chen**

**Supplementary information of analytical results:**

**Table S1** Analytical data of U-Pb isotopes and trace elements of zircon grains

| **Sample** | **Th** | **U** | **Th/U** | **^207^Pb/^206^Pb** | **1σ** | **^207^Pb/^235^U** | **1σ** | **^206^Pb/^238^U** | **1σ** | **^207^Pb/^206^Pb** | **1σ** | **^207^Pb/^235^U** | **1σ** | **^206^Pb/^238^U** | **1σ** |
| --- | --- | --- | --- | --- | --- | --- | --- | --- | --- | --- | --- | --- | --- | --- | --- |
| **Spot NO.** |  |  |  |  |  |  |  |  |  | **Isotopic age (Ma)** | | | | | |
| **LWC-14-16** | arfvedsonite granite | | | | |  |  |  |  |  |  |  |  |  |  |
| LWC-14-16-1 | 179 | 267 | 0.67 | 0.1004 | 0.0027 | 4.0707 | 0.1191 | 0.2948 | 0.0042 | 1631 | 46 | 1648 | 24 | 1666 | 21 |
| LWC-14-16-2 | 214 | 180 | 1.18 | 0.0976 | 0.0028 | 3.8833 | 0.1137 | 0.2914 | 0.0048 | 1589 | 54 | 1610 | 24 | 1649 | 24 |
| LWC-14-16-3 | 390 | 825 | 0.47 | 0.0965 | 0.0025 | 3.5773 | 0.0957 | 0.2668 | 0.0036 | 1558 | 48 | 1545 | 21 | 1524 | 18 |
| LWC-14-16-4 | 535 | 843 | 0.63 | 0.0997 | 0.0029 | 3.6647 | 0.1133 | 0.2641 | 0.0033 | 1620 | 54 | 1564 | 25 | 1511 | 17 |
| LWC-14-16-5 | 1446 | 2422 | 0.60 | 0.1001 | 0.0024 | 3.6997 | 0.0895 | 0.2657 | 0.0036 | 1628 | 46 | 1571 | 19 | 1519 | 19 |
| LWC-14-16-6 | 636 | 631 | 1.01 | 0.1000 | 0.0023 | 3.5351 | 0.0801 | 0.2554 | 0.0037 | 1624 | 43 | 1535 | 18 | 1466 | 19 |
| LWC-14-16-7 | 239 | 427 | 0.56 | 0.0991 | 0.0024 | 3.5629 | 0.0878 | 0.2598 | 0.0040 | 1607 | 45 | 1541 | 20 | 1489 | 21 |
| LWC-14-16-8 | 256 | 508 | 0.50 | 0.0983 | 0.0023 | 3.8589 | 0.0895 | 0.2846 | 0.0046 | 1592 | 44 | 1605 | 19 | 1614 | 23 |
| LWC-14-16-9 | 1036 | 1368 | 0.76 | 0.0971 | 0.0022 | 3.7721 | 0.0871 | 0.2801 | 0.0041 | 1570 | 43 | 1587 | 19 | 1592 | 21 |
| LWC-14-16-10 | 624 | 380 | 1.64 | 0.0977 | 0.0029 | 3.5564 | 0.1146 | 0.2625 | 0.0040 | 1583 | 56 | 1540 | 26 | 1503 | 21 |
| LWC-14-16-11 | 213 | 372 | 0.57 | 0.0999 | 0.0024 | 3.9113 | 0.0983 | 0.2839 | 0.0045 | 1622 | 51 | 1616 | 20 | 1611 | 23 |
| LWC-14-16-12 | 224 | 585 | 0.38 | 0.0993 | 0.0026 | 3.9566 | 0.1004 | 0.2885 | 0.0040 | 1610 | 48 | 1625 | 21 | 1634 | 20 |
| LWC-14-16-13 | 982 | 1858 | 0.53 | 0.1038 | 0.0028 | 3.5602 | 0.0959 | 0.2470 | 0.0033 | 1692 | 50 | 1541 | 21 | 1423 | 17 |
| LWC-14-16-14 | 838 | 1773 | 0.47 | 0.0976 | 0.0029 | 3.6955 | 0.1057 | 0.2719 | 0.0031 | 1589 | 57 | 1570 | 23 | 1550 | 16 |
| LWC-14-16-15 | 768 | 1584 | 0.49 | 0.0973 | 0.0026 | 3.9773 | 0.1060 | 0.2939 | 0.0038 | 1574 | 55 | 1630 | 22 | 1661 | 19 |
| LWC-14-16-16 | 705 | 1461 | 0.48 | 0.0989 | 0.0024 | 3.9146 | 0.0940 | 0.2848 | 0.0031 | 1606 | 46 | 1617 | 19 | 1615 | 16 |
| LWC-14-16-17 | 243 | 365 | 0.66 | 0.0971 | 0.0029 | 3.8131 | 0.1151 | 0.2850 | 0.0048 | 1569 | 56 | 1596 | 24 | 1617 | 24 |
| LWC-14-16-18 | 875 | 630 | 1.39 | 0.0998 | 0.0025 | 3.9027 | 0.1016 | 0.2821 | 0.0043 | 1620 | 46 | 1614 | 21 | 1602 | 22 |
| LWC-14-16-19 | 139 | 353 | 0.39 | 0.1112 | 0.0105 | 4.8475 | 0.5176 | 0.3195 | 0.0118 | 1820 | 173 | 1793 | 90 | 1787 | 58 |
| LWC-14-16-20 | 256 | 559 | 0.46 | 0.1023 | 0.0025 | 3.9199 | 0.0973 | 0.2755 | 0.0037 | 1666 | 45 | 1618 | 20 | 1569 | 19 |
| LWC-14-16-21 | 373 | 266 | 1.41 | 0.1039 | 0.0027 | 3.6074 | 0.0922 | 0.2517 | 0.0037 | 1696 | 44 | 1551 | 20 | 1447 | 19 |
| LWC-14-16-22 | 1662 | 3101 | 0.54 | 0.0992 | 0.0016 | 3.6777 | 0.0632 | 0.2666 | 0.0027 | 1610 | 25 | 1567 | 14 | 1523 | 14 |
| LWC-14-16-23 | 1494 | 3097 | 0.48 | 0.0978 | 0.0016 | 3.5896 | 0.0625 | 0.2642 | 0.0024 | 1583 | 31 | 1547 | 14 | 1511 | 12 |
| **LWC-14-21** | Aegirine-augite granite | | | | | | | |  |  |  |  |  |  |  |
| LWC-14-21-1 | 259 | 443 | 0.58 | 0.1007 | 0.0048 | 3.9577 | 0.1736 | 0.2794 | 0.0052 | 1639 | 89 | 1626 | 36 | 1588 | 26 |
| LWC-14-21-2 | 437 | 585 | 0.75 | 0.0984 | 0.0042 | 3.8661 | 0.1613 | 0.2816 | 0.0048 | 1594 | 81 | 1607 | 34 | 1600 | 24 |
| LWC-14-21-3 | 328 | 631 | 0.52 | 0.0973 | 0.0037 | 3.9188 | 0.1429 | 0.2866 | 0.0044 | 1573 | 72 | 1618 | 30 | 1625 | 22 |
| LWC-14-21-4 | 410 | 793 | 0.52 | 0.0959 | 0.0042 | 3.7663 | 0.1558 | 0.2759 | 0.0069 | 1547 | 83 | 1586 | 33 | 1571 | 35 |
| LWC-14-21-5 | 343 | 972 | 0.35 | 0.1024 | 0.0024 | 3.9716 | 0.0897 | 0.2796 | 0.0029 | 1678 | 43 | 1628 | 18 | 1589 | 14 |
| LWC-14-21-6 | 329 | 845 | 0.39 | 0.1018 | 0.0022 | 3.9002 | 0.0798 | 0.2765 | 0.0027 | 1657 | 40 | 1614 | 17 | 1574 | 14 |
| LWC-14-21-7 | 164 | 177 | 0.92 | 0.1015 | 0.0034 | 3.7417 | 0.1182 | 0.2672 | 0.0038 | 1652 | 66 | 1580 | 25 | 1526 | 19 |
| LWC-14-21-8 | 186 | 314 | 0.59 | 0.1011 | 0.0027 | 3.7112 | 0.1019 | 0.2663 | 0.0034 | 1644 | 49 | 1574 | 22 | 1522 | 17 |
| LWC-14-21-9 | 356 | 729 | 0.49 | 0.0997 | 0.0022 | 3.8455 | 0.0827 | 0.2774 | 0.0029 | 1618 | 41 | 1602 | 17 | 1578 | 15 |
| LWC-14-21-10 | 299 | 545 | 0.55 | 0.0993 | 0.0024 | 3.9865 | 0.0935 | 0.2936 | 0.0063 | 1610 | 45 | 1631 | 19 | 1660 | 31 |
| LWC-14-21-11 | 261 | 448 | 0.58 | 0.0993 | 0.0024 | 3.6811 | 0.0913 | 0.2697 | 0.0036 | 1610 | 45 | 1567 | 20 | 1539 | 18 |
| LWC-14-21-12 | 339 | 306 | 1.11 | 0.0984 | 0.0029 | 3.9938 | 0.1225 | 0.2936 | 0.0041 | 1594 | 54 | 1633 | 25 | 1660 | 21 |
| LWC-14-21-13 | 229 | 491 | 0.47 | 0.0982 | 0.0022 | 3.7541 | 0.0855 | 0.2761 | 0.0033 | 1591 | 41 | 1583 | 18 | 1572 | 17 |
| LWC-14-21-14 | 380 | 627 | 0.61 | 0.0976 | 0.0022 | 3.5379 | 0.0924 | 0.2626 | 0.0043 | 1589 | 43 | 1536 | 21 | 1503 | 22 |
| LWC-14-21-15 | 219 | 420 | 0.52 | 0.0976 | 0.0027 | 3.7507 | 0.1068 | 0.2819 | 0.0050 | 1589 | 54 | 1582 | 23 | 1601 | 25 |
| LWC-14-21-16 | 390 | 625 | 0.62 | 0.0973 | 0.0025 | 3.6314 | 0.0910 | 0.2675 | 0.0033 | 1574 | 47 | 1556 | 20 | 1528 | 17 |
| LWC-14-21-17 | 273 | 362 | 0.75 | 0.0970 | 0.0027 | 3.6787 | 0.0975 | 0.2726 | 0.0030 | 1569 | 51 | 1567 | 21 | 1554 | 15 |
| LWC-14-21-18 | 254 | 421 | 0.60 | 0.0964 | 0.0025 | 3.7508 | 0.1032 | 0.2848 | 0.0060 | 1567 | 50 | 1582 | 22 | 1616 | 30 |
