# Supplementary material for: Geochemistry and tectonic significance of late Paleoproterozoic A-type granites along the southern margin of the North China Craton
Source: Sci Rep. 2020 Jan 9;10:86. doi: 10.1038/s41598-019-56820-1 (PMC6952446; doi:10.1038/s41598-019-56820-1)
Supplement: Supplementary file 2 — Table S2. [file 41598_2019_56820_MOESM2_ESM.docx]

**Geochemistry and tectonic significance of late Paleoproterozoic A-type granites along the southern margin of the North China Craton**

**Yan Wang, Yi-Zeng Yang, Wolfgang Siebel, He Zhang, Yuan-Shuo Zhang, Fukun Chen**

**Supplementary information of analytical results:**

**Table S2** Contents of major and trace elements of the LWZ granite samples

| Sample | HY-14-16 | HY-14-20 | HY-14-21 | HY-14-22 | HY-14-23 | HY-14-25 |
| --- | --- | --- | --- | --- | --- | --- |
| SiO_2_ | 71.1 | 73.0 | 73.4 | 73.2 | 71.5 | 72.6 |
| TiO_2_ | 0.40 | 0.30 | 0.22 | 0.27 | 0.30 | 0.25 |
| Al_2_O_3_ | 12.35 | 11.95 | 12.50 | 11.85 | 12.85 | 12.05 |
| ^T^Fe_2_O_3_ | 4.43 | 3.39 | 1.72 | 3.12 | 3.66 | 3.04 |
| MnO | 0.08 | 0.09 | 0.06 | 0.11 | 0.07 | 0.09 |
| MgO | 0.11 | 0.06 | 0.22 | 0.21 | 0.05 | 0.17 |
| CaO | 1.17 | 1.04 | 1.26 | 1.18 | 1.00 | 0.84 |
| Na_2_O | 3.59 | 3.46 | 3.27 | 2.89 | 3.27 | 3.14 |
| K_2_O | 4.83 | 5.09 | 5.63 | 5.21 | 5.99 | 5.13 |
| P_2_O_5_ | 0.04 | 0.03 | 0.02 | 0.02 | 0.02 | 0.02 |
| LOI | 1.19 | 1.20 | 1.52 | 2.28 | 1.00 | 2.22 |
| Total | 99.33 | 99.65 | 99.87 | 100.37 | 99.74 | 99.58 |
|  |  |  |  |  |  |  |
| FeOtot | 4.02 | 3.08 | 1.56 | 2.83 | 3.32 | 2.76 |
| Mg^#^ | 4.65 | 3.36 | 20.08 | 11.68 | 2.61 | 9.90 |
| FeO_tot_/(FeO_tot_+MgO) | 0.97 | 0.98 | 0.88 | 0.93 | 0.99 | 0.94 |
| A/CNK | 0.93 | 0.91 | 0.91 | 0.95 | 0.94 | 0.98 |
| A/NK | 1.11 | 1.07 | 1.09 | 1.14 | 1.08 | 1.12 |
| Tzr (°C) | 996 | 972 | 937 | 960 | 962 | 972 |
|  |  |  |  |  |  |  |
| Li | 14.33 | 10.25 | 6.94 | 10.53 | 13.73 | 15.74 |
| Be | 3.51 | 5.90 | 2.99 | 3.71 | 7.09 | 4.77 |
| Sc | 6.32 | 4.36 | 3.79 | 4.15 | 4.69 | 2.45 |
| V | 8.63 | 8.38 | 8.28 | 9.13 | 8.99 | - |
| Cr | 5.11 | 4.73 | 3.87 | 5.33 | 4.36 | - |
| Ni | 1.82 | 1.18 | 1.24 | 1.27 | 0.81 | 0.27 |
| Cu | 24.45 | 3.44 | 17.50 | 6.38 | 2.56 | 7.26 |
| Zn | 104.2 | 125.8 | 135.0 | 168.9 | 83.67 | 198.4 |
| Ga | 34.49 | 28.90 | 27.84 | 29.63 | 32.79 | 47.68 |
| Rb | 155.8 | 169.8 | 118.6 | 143.4 | 160.4 | 227.2 |
| Sr | 90.99 | 18.82 | 53.48 | 30.18 | 26.24 | 40.16 |
| Y | 147.8 | 99.04 | 77.54 | 89.53 | 117.3 | 155.5 |
| Zr | 898.9 | 886.9 | 483.7 | 662.7 | 794.1 | 1082 |
| Nb | 131.9 | 100.6 | 71.43 | 97.63 | 109.4 | 141.1 |
| Cs | 1.52 | 1.70 | 0.40 | 4.74 | 1.85 | 1.54 |
| Ba | 192.2 | 135.2 | 201.2 | 110.0 | 119.2 | 187.2 |
| La | 278.3 | 125.3 | 168.6 | 151.3 | 195.7 | 196.8 |
| Ce | 560.4 | 200.9 | 292.2 | 263.0 | 359.6 | 385.6 |
| Pr | 59.89 | 30.98 | 35.41 | 33.80 | 44.54 | 52.32 |
| Nd | 215.5 | 119.2 | 124.6 | 123.8 | 161.9 | 206.4 |
| Sm | 36.20 | 22.52 | 19.76 | 21.39 | 27.63 | 39.52 |
| Eu | 1.44 | 0.92 | 1.09 | 0.95 | 1.12 | 1.68 |
| Gd | 38.30 | 24.04 | 20.88 | 22.35 | 29.58 | 38.72 |
| Tb | 5.57 | 3.59 | 2.89 | 3.16 | 4.28 | 5.31 |
| Dy | 30.65 | 20.18 | 15.09 | 17.91 | 23.71 | 28.80 |
| Ho | 6.19 | 3.98 | 3.01 | 3.50 | 4.66 | 6.35 |
| Er | 17.05 | 11.06 | 8.63 | 10.06 | 12.85 | 17.12 |
| Tm | 2.36 | 1.44 | 1.23 | 1.47 | 1.72 | 2.50 |
| Yb | 14.96 | 9.13 | 8.02 | 9.40 | 11.28 | 15.97 |
| Lu | 2.27 | 1.43 | 1.23 | 1.51 | 1.68 | 2.54 |
| Hf | 24.31 | 23.09 | 13.41 | 18.51 | 21.43 | 32.64 |
| Ta | 6.65 | 5.09 | 3.58 | 5.31 | 5.16 | 7.95 |
| Pb | 20.74 | 33.02 | 35.38 | 46.81 | 17.42 | 44.48 |
| Th | 48.03 | 28.60 | 27.94 | 26.68 | 37.01 | 37.76 |
| U | 5.62 | 6.41 | 2.22 | 3.24 | 3.46 | 5.68 |
| Sr/Y | 0.62 | 0.19 | 0.69 | 0.34 | 0.22 | 0.26 |
| (La/Yb)_N_ | 13.34 | 9.84 | 15.08 | 11.54 | 12.45 | 8.84 |
| Eu/Eu* | 0.12 | 0.12 | 0.16 | 0.13 | 0.12 | 0.13 |
| ΣREE+Y | 2195 | 1152 | 1417 | 1321 | 1618 | 1155 |
| (Zr+Nb+Ce+Y) | 1739 | 1287 | 925 | 1113 | 1380 | 1764 |
