# Supplementary material for: Geochemistry and tectonic significance of late Paleoproterozoic A-type granites along the southern margin of the North China Craton
Source: Sci Rep. 2020 Jan 9;10:86. doi: 10.1038/s41598-019-56820-1 (PMC6952446; doi:10.1038/s41598-019-56820-1)
Supplement: Supplementary file 3 — Table S3. [file 41598_2019_56820_MOESM3_ESM.docx]

**Geochemistry and tectonic significance of late Paleoproterozoic A-type granites along the southern margin of the North China Craton**

**Yan Wang, Yi-Zeng Yang, Wolfgang Siebel, He Zhang, Yuan-Shuo Zhang, Fukun Chen**

**Supplementary information of analytical results:**

**Table S3** Sr, Nd and Pb isotopic composition of the LWZ granite samples

| **Sample** | **Rb** | **Sr** | **^87^Rb/^86^Sr** | **^87^Sr/^86^Sr** | **±2σ_m_** | **(^87^Sr/^86^Sr)_t_** | **Sm** | **Nd** | **^147^Sm/^144^Nd** | **^143^Nd/^144^Nd** | **±2σ_m_** | **T_DM2_** | **(^143^Nd/^144^Nd)_i_** | **ε_Nd_(t)** |
| --- | --- | --- | --- | --- | --- | --- | --- | --- | --- | --- | --- | --- | --- | --- |
|  | **(ppm)** | **(ppm)** |  |  |  |  | **(ppm)** | **(ppm)** |  |  |  | **(Ga)** |  |  |
| LWC-14-16 | 141.7 | 71.45 | 5.804 | 0.825068 | 0.000015 | 0.69169 | 42.91 | 286.4 | 0.0906 | 0.511514 | 0.000013 | 2.57 | 0.510561 | -0.2 |
| LWC-14-20 | 169.8 | 18.82 | 27.51 | 1.262082 | 0.000015 | 0.62990 | 32.91 | 163.5 | 0.1217 | 0.511581 | 0.000013 | 2.50 | 0.510301 | -5.2 |
| LWC-14-21 | 118.6 | 53.48 | 6.512 | 0.864709 | 0.000014 | 0.71507 | 27.05 | 164.7 | 0.0993 | 0.511375 | 0.000015 | 2.80 | 0.510331 | -4.7 |
| LWC-14-22 | 143.4 | 30.18 | 14.01 | 0.903144 | 0.000012 | 0.58135 | 28.81 | 159.2 | 0.1094 | 0.511451 | 0.000010 | 2.69 | 0.510300 | -5.3 |
| LWC-14-23 | 160.4 | 26.24 | 18.15 | 0.978198 | 0.000013 | 0.56108 | 37.23 | 212.8 | 0.1058 | 0.511438 | 0.000009 | 2.70 | 0.510326 | -4.8 |
| LWC-14-25 | 227.2 | 40.16 | 16.71 | 0.922197 | 0.000014 | 0.53816 | 27.55 | 138.5 | 0.1202 | 0.511597 | 0.000010 | 2.47 | 0.510332 | -4.6 |

| **Sample** | **^206^Pb/^204^Pb** | **±2σ_m_** | **^207^Pb/^204^Pb** | **±2σ_m_** | **^208^Pb/^204^Pb** | **±2σ_m_** | **^206^Pb/^204^Pb**  **(t)** | **^207^Pb/^204^Pb**  **(t)** | **^208^Pb/^204^Pb**  **(t)** |
| --- | --- | --- | --- | --- | --- | --- | --- | --- | --- |
| LWC-14-16 | 20.033 | 0.011 | 15.729 | 0.011 | 48.281 | 0.011 | 14.421 | 15.175 | 33.706 |
| LWC-14-20 | 18.609 | 0.009 | 15.604 | 0.009 | 40.428 | 0.009 | 15.034 | 15.251 | 35.580 |
| LWC-14-21 | 18.518 | 0.010 | 15.588 | 0.010 | 41.373 | 0.010 | 17.349 | 15.472 | 35.305 |
| LWC-14-22 | 18.001 | 0.010 | 15.545 | 0.009 | 39.517 | 0.011 | 16.753 | 15.422 | 36.394 |
| LWC-14-23 | 18.970 | 0.009 | 15.631 | 0.010 | 46.042 | 0.009 | 15.021 | 15.241 | 33.206 |
| LWC-14-25 | 18.318 | 0.009 | 15.576 | 0.009 | 40.906 | 0.009 | 15.961 | 15.343 | 36.145 |
