# Supplementary material for: Geochemistry and tectonic significance of late Paleoproterozoic A-type granites along the southern margin of the North China Craton
Source: Sci Rep. 2020 Jan 9;10:86. doi: 10.1038/s41598-019-56820-1 (PMC6952446; doi:10.1038/s41598-019-56820-1)
Supplement: Supplementary file 4 — Table S4. [file 41598_2019_56820_MOESM4_ESM.docx]

**Geochemistry and tectonic significance of late Paleoproterozoic A-type granites along the southern margin of the North China Craton**

**Yan Wang, Yi-Zeng Yang, Wolfgang Siebel, He Zhang, Yuan-Shuo Zhang, Fukun Chen**

**Supplementary information of analytical methods used in this study:**

**Table S4** Analytical methods

Analytical methods

For whole-rock geochemical analyses, rock samples were crushed into powders <200 mesh. Major element compositions were analyzed using X-ray fluorescence (ALS Minerals-ALS Chemex, Guangzhou, China). Loss on ignition (LOI) was determined by gravimetric methods using an electronic balance. Analytical uncertainties of major elements were better than 1%. From repeated sample runs, the reproducibility of major-element concentrations was estimated to be better than 5%. Trace element abundances were determined by inductively coupled plasma mass spectrometry [Elan^®^ 6100 DRC ICP-MS; Key Laboratory of Crust–Mantle Materials and Environments, University and Technology of China (USTC), Hefei, Anhui, China]. Approximately 50 mg of bulk-rock powder was weighed into a Teflon bomb for the analyses, and Rh solution was used as an internal standard. Analytical uncertainties were generally better than ±5% for most trace elements.

Zircon grains were obtained from crushed rocks by standard mineral separation techniques and were handpicked under a binocular microscope. The grains were mounted on adhesive tape and covered with epoxy resin. Then, the grains were polished to approximately half their thickness and were prepared for cathodoluminescence (CL) imaging to investigate their internal structure. U-Pb zircon dating was performed using laser ablation inductively coupled plasma mass spectrometry (LA-ICP-MS; Agilent 7700E ICP-MS equipped with a 193-nm ArF-excimer laser) at the Key Laboratory of Crust–Mantle Materials and Environments, USTC. The diameter of the laser ablation pit was 32 μm, and the laser frequency was 10 Hz. Each analysis incorporated ~20 s of background acquisition (gas blank) followed by 40 s of data acquisition from the sample and another 20 s of background acquisition. After every four samples, one analysis of standard zircon 91500 to correct the time-dependent drift of sensitivity and mass discrimination. National Institute of Standards and Technology (NIST) Standard Reference Material (SRM) 610 was analyzed twice after every 10 sample spots for calculating concentrations of the rare earth elements (REE) U, Th, and Pb. Off-line evaluation and integration of signal to background, time-drift correction, quantitation calibration for trace element analyses and U-Pb dating, and common lead correction were processed using ICPMS Data Cal software^1^. Concordia diagrams were created and U-Pb age calculations were performed using Isoplot software^2^. All errors are quoted as 2σ.

Whole-rock Sr-Nd-Pb isotopic analyses were performed at the Laboratory for Radiogenic Isotope Geochemistry at USTC. Following sample decomposition, parent and daughter nuclides were isolated from each other using standard chromatographic separation techniques. Rb, Sr, and the light rare-earth elements (LREE) were isolated on quartz columns by conventional ion-exchange chromatography on a 5-ml resin bed of AG 50W-X12 (200–400 mesh). Nd and Sm were separated from other REE on quartz columns using 1.7 ml Teflon powder coated with di(2-ethylhexyl) orthophosphoric acid (HDEHP) as the cation exchange medium. Measurements were performed using a Finnigan MAT-262 mass spectrometer. Sr was loaded with a Ta-HF activator on preconditioned Ta filaments, and Nd was loaded as a phosphate on preconditioned Re filaments. The ratios of Sr and Nd isotope were corrected for mass fractionation relative to ^86^Sr/^88^Sr = 0.1194 and ^146^Nd/^144^Nd = 0.7219, respectively. NIST SRM 987 and La Jolla standard solution were used as standards for Sr and Nd analyses, respectively. Precision of the measured Nd isotopic ratios was better than 0.003%. Pb was separated by anion exchange chromatography with diluted HBr acid as the eluent.

**References**

1. Liu, Y., Hu, Z., Gao, S., Günther, D., Xu, J., Gao, C., Chen, H. In situ analysis of major and trace elements of anhydrous minerals by LA-ICP-MS without applying an internal standard. Chem. Geol. 257, 34-43 (2008).

2. Ludwig, K.R. Isoplot 3.70: a geochronological toolkit for Microsoft Excel. Spec. Publ. 4. Berkeley, CA, Berkeley Geochronology Center (2009).
