# Supplementary material for: Geochemistry and tectonic significance of late Paleoproterozoic A-type granites along the southern margin of the North China Craton
Source: Sci Rep. 2020 Jan 9;10:86. doi: 10.1038/s41598-019-56820-1 (PMC6952446; doi:10.1038/s41598-019-56820-1)
Supplement: Supplementary file 5 — Table S5. [file 41598_2019_56820_MOESM5_ESM.docx]

**Geochemistry and tectonic significance of late Paleoproterozoic A-type granites along the southern margin of the North China Craton**

**Yan Wang, Yi-Zeng Yang, Wolfgang Siebel, He Zhang, Yuan-Shuo Zhang, Fukun Chen**

**Supplementary information of reported zircon age data:**

**Table S5** Summary of zircon U-Pb ages of A-type granites along the southern margin of the NCC

| **Pluton**  **Name** | **Area (km^2^)** | **Rock type** | **Formation age (Ma)** | **Analytic technique** | **Data source** |
| --- | --- | --- | --- | --- | --- |
|  |  |  |  |  |  |
| Guijiayu | 13 | Monzogranite | 1802 ±27 | LA-ICP-MS | Deng et al., 2016 |
|  |  |  | 1803 ±25 | LA-ICP-MS | Deng et al., 2016 |
|  |  |  | 1797 ±28 | LA-ICP-MS | Deng et al., 2016 |
| Luoning | 0.5 | Granite | 1786 ±7 | SIMS | Cui et al., 2012 |
| Motianzhai | 4.0 | Biotite granite | 1797 ±14 | SHRIMP | Zhao and Zhou, 2009 |
| Shicheng | 55 | Biotite granite | 1743 ±14 | SHRIMP | Zhao and Zhou, 2009 |
| Longwang- zhuang | 140 | Arfvedsonite syenogranite | 1637 ±33 | TIMS | Lu et al., 2003 |
|  |  | Arfvedsonite syenogranite | 1625 ±16 | SHRIMP | Lu et al., 2003 |
|  |  | Biotite granite | 1600 ±20 | LA-ICP-MS | Bao et al., 2011 |
|  |  | Sodic ferrogedrite | 1616 ±20 | LA-ICP-MS | Wang et al., 2013 |
|  |  | Arfvedsonite syenogranite | 1601 ±10 | LA-ICP-MS | This study |
|  |  | Syenite granite | 1609 ±18 | LA-ICP-MS | This study |
| Maping | 2.5 | Granite porphyry | 1600 ±24 | LA-ICP-MS | Deng et al., 2015 |
|  |  |  | 1583 ±28 | LA-ICP-MS | Deng et al., 2015 |
| Zhangjiaping | 46 | Biotite granite | 1526 ±17 | LA-ICP-MS | Deng et al., 2016 |
|  |  | Biotite-hornblende granite | 1532 ±16 | LA-ICP-MS | Deng et al., 2016 |
|  |  | K-feldspar granite | 1500 ±13 | LA-ICP-MS | Deng et al. 2016 |
|  |  | Biotite monzogranite | 1508 ±55 | LA-ICP-MS | Deng et al. 2016 |
